# Prevalence and factors associated with undernutrition among HIV-positive children aged 6 months to 12 years attending antiretroviral treatment clinics in Bushenyi District, Uganda: a cross-sectional study

**DOI:** 10.1186/s40795-026-01298-0

**Published:** 2026-03-18

**Authors:** Abdiwahid Mohamed Ahmed, Martin Nduwimana, Jolly Nankunda, Elfeky Walyeldin, Ahmed Hassan Mohamoud, Mohamed Hussein Hassan, Ahmed M. Ali Mohamed, Abbas Hussein Musse, Liban Osman Isse, Melvis Bernis Maren

**Affiliations:** 1 Department of Paediatrics, Faculty of Clinical Medicine and Dentistry, Kampala International University- Western Campus, Bushenyi, Uganda; 2 Department of Paediatrics and Child Health, Makerere University College of Health Sciences, Kampala, Uganda; 3 Department of Paediatrics and Child Health, University of Bahri, Khartoum, Sudan

**Keywords:** Human immune deficiency virus, Undernutrition, Prevalence, Factors associated

## Abstract

**Background:**

Undernutrition remains a major public health concern among HIV-positive children. Despite advancements in antiretroviral therapy (ART) effectiveness in managing HIV, these children still face nutritional challenges that impact their overall health. This study sought to determine the prevalence and identify the factors associated with undernutrition among HIV-positive children attending ART clinics in Bushenyi District, western Uganda.

**Methods:**

Multi-facility based cross-sectional and analytical study was conducted among HIV-positive children aged 6 months to 12 years at five ART clinics in Bushenyi District. The study carried out from November, 2024 to February, 2025, Using consecutive sampling method. Data on socio-demographic characteristics, factors associated were collected using structured questionnaires and anthropometric measurements and analyzed using STATA Version 14.2. A bivariable and multivariable binary logistic regression analysis was performed to assess factors associated with undernutrition among HIV-positive children.

**Results:**

Out of 385 children studied, 177/385 (45.97%) had undernutrition. The prevalence of stunting (36.62%), underweight (24.42%), and wasting (10.91%) being the most common forms, Key predictors of undernutrition included low caregiver education (aOR = 4.54{95% CI: 2.75–8.48}, p-value < 0.001), low household income (aOR = 9.22 {95% CI:3.15–26.93}, p-value < 0.001), low birth weight (aOR = 3.48 {95% CI:1.38–8.76}, p-value = 0.008), high viral load (aOR = 7.43 {95% CI:2.37–23.34}, p-value = 0.001), low CD4 count (aOR = 5.69 {95% CI: 1.81–17.90}, p-value = 0.003), ART duration < 12 months (aOR = 7.80 {95% CI: 2.33–26.10}, p-value = 0.001), missed ART doses > 5 doses (aOR = 8.45 {95% CI: 2.32–30.74}, p-value = 0.001), and opportunistic infections (aOR = 8.26 {95% CI:3.70–18.44}, p-value < 0.001).

**Conclusion:**

The prevalence of undernutrition was high. With significant rates of stunting, underweight, and wasting. Sociodemographic and clinical factors, including caregiver education, household income, ART adherence, and viral load and opportunistic infection, also played critical roles in undernutrition.

**Supplementary Information:**

The online version contains supplementary material available at 10.1186/s40795-026-01298-0.

## Introduction

Undernutrition and HIV remain two of the most pressing global health challenges, particularly in sub-Saharan Africa, which bears the greatest burden of both conditions. More than 90% of the 2. 84 million HIV-positive children under the age of 19 worldwide reside in sub-Saharan Africa [1]. Simultaneously, the region accounts for the highest prevalence of childhood undernutrition, with an estimated 49 million stunted and 149 million wasted children, representing over 90% of the global burden [1–4]. In sub-Saharan Africa, the prevalence of wasting and stunting among children can reach up to 10% and 32%, respectively [1–4].

HIV infection and undernutrition are closely interlinked, creating a vicious cycle in which each condition exacerbates the other. HIV compromises the immune system through chronic inflammation and opportunistic infections, increasing metabolic demands and impairing nutrient absorption [1]. Inadequate intake of energy, protein, and essential micronutrients-such as iron, zinc, and vitamin A-further weakens immune defense, delays recovery, and worsens disease progression [1, 5]. Antiretroviral therapy (ART), while essential for improving survival, can also contribute to nutritional complications through side effects such as nausea, vomiting, and changes in lipid metabolism or bone mineral density, particularly in the early stages of treatment [1]. In Uganda, the most commonly prescribed regimens-TDF/3TC/DTG, ABC/3TC/DTG, and AZT/3TC/DTG-may have variable metabolic impacts that influence nutritional outcomes.

Despite significant progress in ART coverage and child health programs, undernutrition remains a major concern among HIV-positive children. Household food insecurity, low caregiver education, poverty, and limited access to healthcare are known to influence both HIV progression and nutritional outcomes [1, 6, 7]. Furthermore, while risk factors are well documented, few studies have explored protective factors such as caregiver education, community-based nutrition counseling, or support programs that enhance resilience against undernutrition in HIV-affected households. Addressing both vulnerability and resilience factors is crucial for designing sustainable interventions.

Several studies in sub-Saharan Africa have reported high rates of malnutrition among children living with HIV. For example, a regional meta-analysis found pooled prevalence of stunting (35. 9%), underweight (23. 0%), and wasting (23. 0%) [1]. In Uganda, a study at Hoima Regional Referral Hospital reported a 28. 6% prevalence of malnutrition among HIV-positive children under five years of age [8]. However, most previous studies have focused primarily on children under five, leaving older children—who experience continued nutritional vulnerability due to ART side effects, school feeding patterns, and growth demands largely understudied.

Uganda has implemented several national strategies to address the nutritional needs of children living with HIV, including the Uganda Nutrition Action Plan II (UNAP II, 2020–2025), the Integrated Management of Acute Malnutrition (IMAM) program, and Prevention of Mother-to-Child Transmission (PMTCT) initiatives that incorporate nutritional supplementation and counseling. However, data on the nutritional outcomes of HIV-positive children aged 5–12 years remain limited, making it difficult to assess the effectiveness of these interventions across all age groups.

Given this gap, this study focuses on HIV-positive children aged 6 months to 12 years—a broader age range than most existing research—to capture patterns of nutritional status across infancy, early childhood, and middle childhood. This approach provides a more comprehensive understanding of the factors associated with undernutrition across developmental stages.

Therefore, this study does not aim to establish causality but rather to determine the prevalence and identify modifiable factors associated with undernutrition among HIV-positive children attending ART clinics in Bushenyi District, western Uganda. By doing so, the study contributes evidence to guide targeted interventions and inform Uganda’s on-going efforts to integrate nutritional support within pediatric HIV care programs.

## Materials and methods

### Study design and setting of the study

This was a multicentre health facility-based cross sectional, descriptive and analytical study conducted in selected ART clinics in Bushenyi district. Bushenyi District is located in western Uganda, within the Ankole sub-region. The total area of the district is 942. 3 square kilometers, with a total population of 251, 400 and a population density of 266. 8 per square kilometer. HIV-positive children were enrolled at Bushenyi Health Centre IV (BHC), Kampala International University Teaching Hospital (KIU-TH), Ishaka Adventist Hospital (IAH), Kyabugimbi Health Centre IV (KHC), and Comboni Hospital (CH). These health facilities are located in Bushenyi District, with three in the urban setting and two in the rural setting.

### Study participants

Included all HIV-positive children aged 6 months to 12 years who presented to the selected health facilities during the study period, were on ART, whose parents/guardians provided consent, and who provided assent (where applicable). Children who met the inclusion criteria but with chronic medical conditions unrelated to HIV, such as congenital anomalies, cerebral palsy, or genetic syndromes, that could independently affect growth and nutritional outcomes, as well as those whose caregivers were unwilling to participate, were excluded.

This study was conducted for a period of three months, from November 2024 to February 2025.

### Sampling technique

Five health facilities were selected using stratified sampling, with three facilities chosen from urban settings (BHC, KIU-TH, and IAH) and two from rural settings (KHC and CH). Selecting children from both rural and urban settings enhanced the generalizability of the findings, ensuring a sample representative of the entire district.

Stratified sampling is a sampling method used by researchers to divide a bigger population into subgroups or strata, which can then be further used to draw samples using a random sampling method. The stratified sampling technique is useful in ensuring that every subgroup, or stratum, within the population is adequately represented in the sample.

The number of participants selected from each HAART clinic was determined using proportional sampling, based on the total number of HIV-infected children enrolled at each facility. Three months prior to the survey, a total of 403 HIV-infected children were registered across the five HAART clinics, with the following distribution: KIU-TH (20), Ishaka Adventist Hospital (114), Bushenyi HC IV (50), St. Daniel Comboni Hospital (154), and Kyabugimbi HC IV (65). Based on the proportionate distribution of the sample, 19 participants were recruited from KIU-TH, 109 from Ishaka Adventist Hospital, 48 from Bushenyi HC IV, 147 from St. Daniel Comboni Hospital, and 62 from Kyabugimbi HC IV. Participants were consecutively enrolled at each HAART clinic until the allocated sample size was attained.

### Variables and data sources

Eligible participants have appropriately consented/ assented, and information on socio-demographics, and other medical factors was collected by the Principal investigator and trained research assistants using pretested interviewer-administered structured questionnaires. Body weight was measured using a digital scale, and height was measured using a stadiometer. For children unable to stand, length was determined using a measuring board. The weight, length, and BMI of each child were plotted onto WHO growth charts to determine their nutritional status, utilizing tools available through the WHO Growth Standards platform.

### Sample size calculation

For objective one, the Kish-Leslie Formula was used to determine the Sample size for this study, using the estimated prevalence of 49. 68% based a study from east Africa [9], and the calculated sample size was 385 participants.

For objective two, the openEpi online (n.d) sample size calculator (https://www.openepi.com/SampleSize/SSCohort. htm accessed on June 4, 2024) was used. Based on findings from a study conducted in Rakai, Uganda—which reported higher levels of malnutrition among orphaned children (47%) compared to those with both parents (28%) [10], a sample size of 224 was calculated using the Fleiss method, with a power of 80% and a 95% confidence level.

The larger sample size obtained from objective one (385participants) was used.

### Data quality control

Before the commencement of data collection, each questionnaire underwent pretesting to ensure reliability and validity. The interviews were conducted in Runyankole, the local language, to enhance participant comprehension and engagement.

All research instruments were regularly calibrated and validated to maintain precision. To ensure ethical adherence and the proper use of data collection tools, the principal investigator provided regular supervision of research assistants. The primary investigator also reviewed and corrected the questionnaires during the data collection process. Comprehensive history taking and physical examinations were conducted for all participants. To ensure accuracy in measurements, two weight and height measurements were taken, with a third measurement conducted if discrepancies were observed between the first two.

### Data analysis

**Fig. 1:**
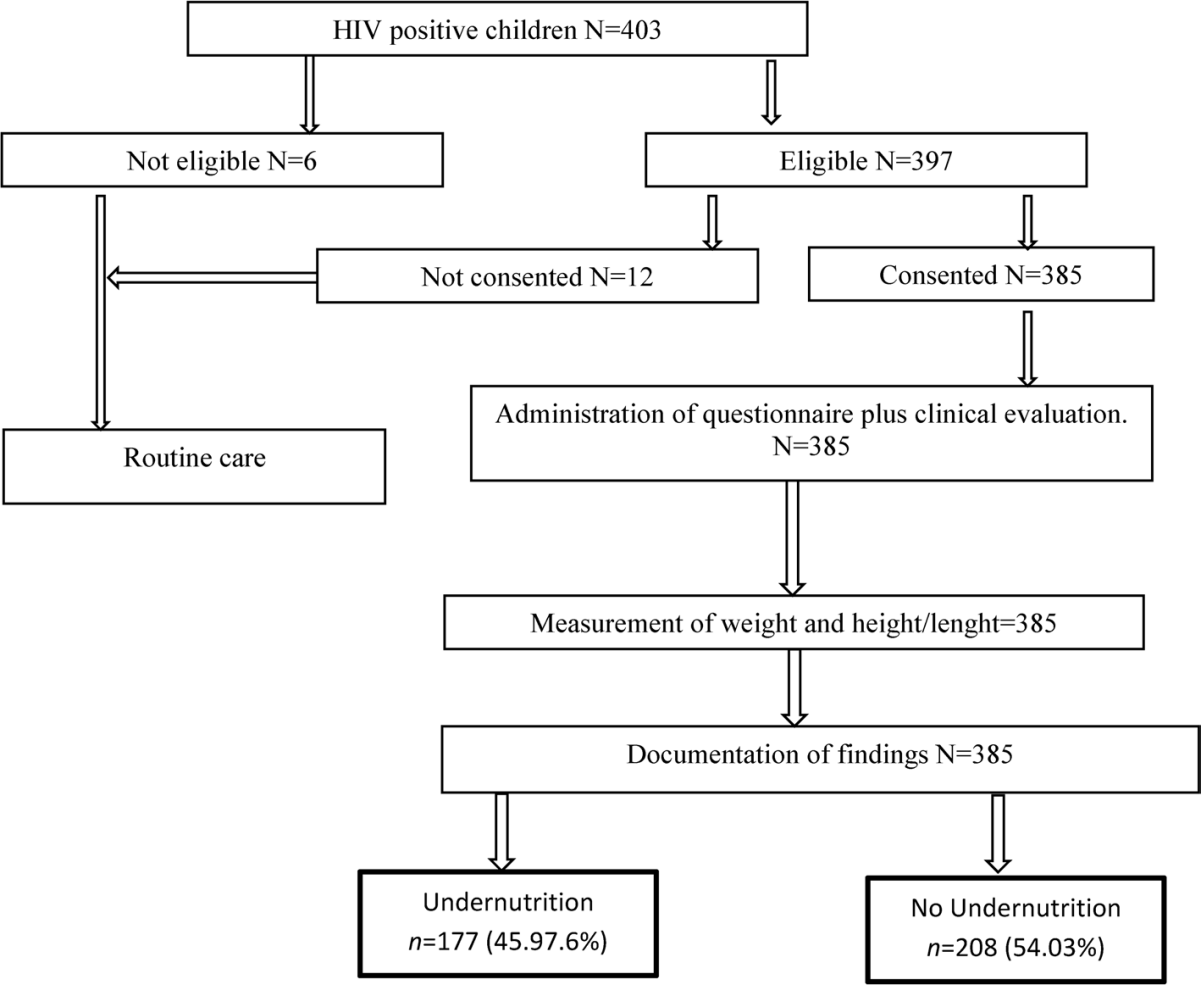
Study flow chart of participants

Data analysis for this study was performed using STATA version 14. 2, structured to address two primary objectives. The prevalence of undernutrition was calculated as a fraction of participants with undernutrition against all participants enrolled in the study and expressed as frequency and percentage. Binary logistic regression was performed to identify factors associated with undernutrition. Odds ratios (ORs), p-values, and 95% confidence intervals (CIs) were reported. Variables with p-values less than 0. 2 in the bivariable analysis were included in the multivariable model. In the final model, a p-value of less than 0. 05 was considered statistically significant (Fig. 1).

## Results

### Characteristics of study participants

**Fig. 2:**
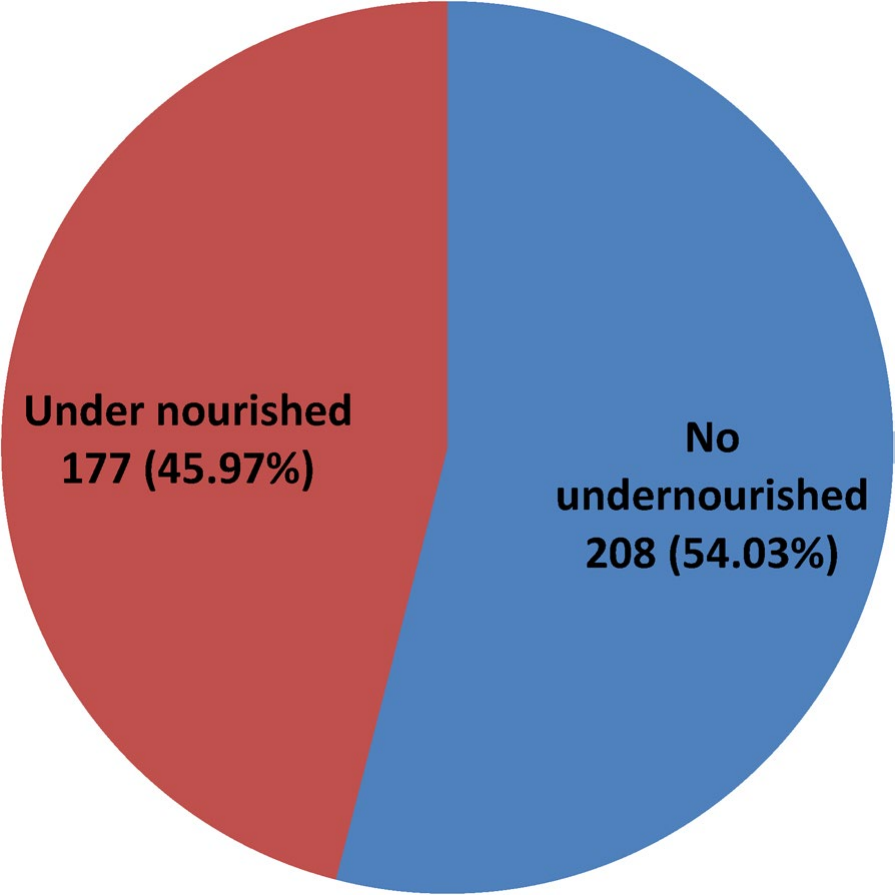
Prevalence of Undernutrition among HIV positive children. Among the 385 participants included in the study, 177 were classified as undernourished, yielding a prevalence of 45.97% (95% CI:41.03%–50.99%). Wasting was identified in 42 participants (10.91%; 95% CI: 8.15%–14.45%), underweight in 94 participants (24.42%; 95% CI:20.37%–28.98%), stunting in 141 participants (36.62%; 95% CI31.94%–41.58%), and WaSt in 18 participants (4.68%; 95% CI:2.97%–7.26%)

A total of 385 children receiving HIV care at five health facilities participated in this study, and nutritional assessment was done (Fig. 2). The participants’ ages ranged from 6 months to 12 years, with the majority aged between 2 and 12 years (82.6%), with an almost equal male-to-female ratio: Male, 195 (50.65%), Female: 190 (49.35%). The majorities of caregivers were young adults 147 (38.18%), and most had at least primary level of education 167 (43.38%). Informal employment was the most common occupation 161 (41.82%), with a nearly equal urban-rural distribution: Urban: 193 (50.13%), Rural: 192 (49.87%). Almost a third had five or more children, and most families earned below 500, 000 UGX 165 (42. 86%). Mothers were the primary caregivers 196 (50.91%), followed by grandparents 124 (32.21%). More than half of the children were second to fourth-born 211 (54.81%), (Table 1).

**Table 1:** Sociodemographic characteristics of undernutrition among HIV-positive children (N=385)

Characteristic	Frequency (*n*)	Percentage (%)
Age Category (yrs.)		
< 2	67	17.40
2–6	158	41.04
6–12	160	41.56
Sex of the Child		
Male	195	50.65
Female	190	49.35
Mother’s Age Category (yrs.)		
Teenager (13–19)	31	8.05
Young Adult (20–29)	147	38.18
Adult (30–39)	137	35.58
Older Adult (40+)	70	18.18
Caregiver’s Education Level		
None	72	18.70
Primary	167	43.38
Secondary	146	37.92
Caregiver’s Occupation		
Formal Employee	117	30.39
Informal Employee	161	41.82
Unemployed	107	27.79
Residence		
Urban	193	50.13
Rural	192	49.87
Total Number of Living Children		
1	82	21.30
2	85	22.08
3	62	16.10
4	80	20.78
5+	76	19.74
Monthly Family Income (UGX)		
< 200,000	130	33.77
200,000–500,000	165	42.86
> 500,000	90	23.38
Caregiver Relationship		
Mother	196	50.91
Grandparent	124	32.21
Other	65	16.88
Birth Order		
1st Child	64	16.62
2nd–4th Child	211	54.81
5th+ Child	110	28.57

Additionally, most children had a birth weight of at least 2. 5 kg 289 (75.06%). The majority had a history of hospital admission 230 (59.74%). Regarding viral load, most had values between 40 and 1,000 copies/ml 178 (46.23%). More than half of the children had CD4 counts of at least 350 cells/mm³ 216 (56.10%), Stage 1 HIV was the most common classification (317,82.34%), while Stage 3&4 were the least frequent 23 (5.98%). ART regimens were distributed across TDF/3TC/ DTG 135 (35.06%), ABC/3TC/DTG 153 (39.74%), and AZT/3TC/ DTG 97 (25.19%) without significant dominance. Most had been on ART for over 36 months 230 (59.74%). Missed ART doses were reported, with a substantial proportion having missed fewer than two. Cotrimoxazole use was prevalent 219 (56.88%), and opportunistic infections were noted in over a third of participants 157 (40.78%), (Table 2).

**Table 2:** Clinical characteristics of undernutrition among HIV-positive children (*N* = 385)

Characteristic	Frequency (*n*)	Percentage (%)
Birth Weight		
< 2.5 kg	96	24.94
≥ 2.5 kg	289	75.06
History of Hospital Admission		
Yes	230	59.74
No	155	40.26
Recent Viral Load (copies/ml)		
< 40 copies/ml	139	36.10
< 1000 copies/ml	178	46.23
> 1000 copies/ml	68	17.66
CD4 Count (cells/mm³)		
< 200	61	15.84
200–349	108	28.05
≥ 350	216	56.10
HIV Clinical Stage		
Stage 1	317	82.34
Stage 2	45	11.69
Stage 3&4	23	5.98
ART Regimen		
TDF/3TC/ DTG	135	35.06
ABC/3TC/DTG	153	39.74
AZT/3TC/ DTG	97	25.19
Duration on ART		
< 12 months	60	15.58
12–36 months	95	24.68
> 36 months	230	59.74
Missed ART Doses (Last Month)		
< 2 doses	98	25.45
2–5 doses	51	13.25
> 5 doses	50	12.99
None	186	48.31
Cotrimoxazole Use		
Yes	219	56.88
No	166	43.12
Opportunistic Infections		
Yes	157	40.78
No	228	59.22

### Prevalence of undernutrition among HIV positive children

Among the 385 participants included in the study, 177 were classified as undernourished, yielding a prevalence of 45.97% (95% CI: 41.03%–50.99%). Wasting was identified in 42 participants (10.91%;95% CI: 8.15%–14.45%), underweight in 94 participants (24.42%; 95% CI:20.37%–28.98%), stunting in 141 participants (36.62%; 95% CI:31.94%–41.58%), and WaSt in 18 participants (4.68%; 95% CI: 2.97%–7.26%), (Fig. 2).

### Factors associated with Undernutrition among HIV-Positive children

At bivariable analysis results indicated that three socio-demographic factors were significantly associated with undernutrition (*P* ≤ 0.2). These factors included Children whose mothers had no formal education (cOR = 7.42; 95% CI:3.94–13.97; *p* < 0.001) were over seven times more likely to be undernourished compared to those formal education, and those with primary education (cOR = 3.57; 95% CI:2.20–5.79; *p* < 0.001) also were over seven times more likely to be undernourished compared to those whose mothers had secondary education. Children living in rural areas (OR = 4.17; 95% CI: 2.72–6.39; *p* < 0.001) were four times more likely to be undernourished compared to those in urban settings. Children from households earning less than 200,000 UGX per month (cOR = 10.86; 95% CI: 5.69–20.71; *p* < 0.001) had over ten times the odds of being undernourished compared to those from households earning more than 200,000 UGX (cOR = 2.53; 95% CI:1.39–4.64; *p* = 0.003), (Table 3).

**Table 3:** Bivariate analysis of sociodemographic factors associated with undernutrition among HIV-positive children (*N* = 385)

Characteristic	No Undernutrition *n* (%)	Undernutrition *n* (%)	OR (95% CI)	*p*-value
Age Category (Yrs)				
< 2	33 (49.25)	34 (50.75)	1.11 (0.63–1.96)	0.719
2–6	92 (58.23)	66 (41.77)	0.77 (0.50–1.20)	0.255
6–12	83 (51.88)	77 (48.13)	Ref	-
Sex of the Child				
Male	111 (56.92)	84 (43.08)	Ref	-
Female	97 (51.05)	93 (48.95)	1.27 (0.85–1.89)	0.248
Mother’s Education Level				
None	21 (29.17)	51 (70.83)	7.42 (3.94–13.97)	**< 0.001**
Primary	77 (46.11)	90 (53.89)	3.57 (2.20–5.79)	**< 0.001**
Secondary	110 (75.34)	36 (24.66)	Ref	**-**
Mother’s Occupation				
Formal Employee	60 (51.28)	57 (48.72)	Ref	-
Informal Employee	91 (56.52)	70 (43.48)	0.81 (0.50–1.31)	0.387
Unemployed	57 (53.27)	50 (46.73)	0.92 (0.55–1.56)	0.766
Residence				
Urban	137 (70.98)	56 (29.02)	Ref	**-**
Rural	71 (36.98)	121 (63.02)	4.17 (2.72–6.39)	**< 0.001**
Monthly Family Income (UGX)				
< 200,000	35 (26.92)	95 (73.08)	10.86 (5.69–20.71)	**< 0.001**
200,000–500,000	101 (61.21)	64 (38.79)	2.53 (1.39–4.64)	**0.003**
> 500,000	72 (80.00)	18 (20.00)	Ref	-
Caregiver Relationship				
Mother	105 (53.57)	91 (46.43)	Ref	-
Grandparent	66 (53.23)	58 (46.77)	1.01 (0.65–1.59)	0.952
Other	37 (56.92)	28 (43.08)	0.87 (0.50–1.54)	0.638
Birth Order				
1st Child	32 (50.00)	32 (50.00)	Ref	-
2nd–4th Child	110 (52.13)	101 (47.87)	0.92 (0.52–1.61)	0.765
5th+ Child	66 (60.00)	44 (40.00)	0.67 (0.36–1.24)	0.201

* p-value (< 0. 2). *Ref* Reference category, *OR* Odds Ratio, *CI* Confidence Interval

Clinical factors significantly associated with undernutrition (*p* < 0.2) included Children born with a low birth weight (< 2.5 kg) (cOR: 3.53 (95% CI:2.16–5.77), *p* < 0.001) were 3.53 times more likely to be undernourished compared to those children with birth weight ≥ 2.5 kg, Children who had ever been admitted (cOR:1.79 (95% CI:1.18–2.72), *p* = 0.006) were nearly 1.8 times more likely to be undernourished compared to those never admitted, Children with > 1000 copies/ml (cOR:10.09 (95% CI:5.15–19.79), *p* < 0.001) had 10 times greater odds of being undernourished compared to those with viral suppression, those Children with lower CD4 counts, particularly < 200 (cOR:20.53 (95% CI: 8.84–47.66), *p* < 0.001) had 20 times more likely of being undernourished compared to those with their CD4 ≥ 350, HIV clinical Stage 3&4 (cOR = 2.64 (95% CI:0.99–7.01), *p* = 0.118) had 2.64 times more likely of being undernourished compared to those with HIV clinical Stage1, regarding ART duration < 12 months (cOR = 14.70 (95% CI: 6.84–31.59), *p* < 0.001) were 14.70 times more likely of be undernourished compared to those with ART duration > 36 months, Children with opportunistic infections (cOR = 14.11 (95% CI: 8.54–23.29), *p* < 0.001) had a 14.11-fold increased likelihood of being undernourished compared to children who did not experience opportunistic infections, (Table 4).

**Table 4:** Bivariate analysis of clinical factors associated with undernutrition among HIV-positive children (*N* = 385)

Characteristic	No Undernutrition *n* (%)	Undernutrition *n* (%)	OR (95% CI)	*p*-value
Birth Weight				
< 2.5 kg	30 (31.25)	66 (68.75)	3.53 (2.16–5.77)	**< 0.001**
≥ 2.5 kg	178 (61.59)	111 (38.41)	Ref	**-**
Child Ever Admitted				
Yes	111 (48.26)	119 (51.74)	1.79 (1.18–2.72)	**0.006**
No	97 (62.58)	58 (37.42)	Ref	**-**
Recent Viral Load				
< 40)	109 (78.42)	30 (21.58)	Ref	**-**
< 1000	81 (45.51)	97 (54.49)	4.35 (2.64–7.18)	**< 0.001**
> 1000	18 (26.47)	50 (73.53)	10.09 (5.15–19.79)	**< 0.001**
CD4 Count				
< 200	7 (11.48)	54 (88.52)	20.53 (8.84–47.66)	**< 0.001**
200–349	44 (40.74)	64 (59.26)	3.87 (2.38–6.30)	**< 0.001**
≥ 350	157 (72.69)	59 (27.31)	Ref	**-**
HIV Clinical Stage				
Stage 1	170 (53.63)	147 (46.37)	Ref	**-**
Stage 2	31 (68.89)	14 (31.11)	0.50 (0.32–1.50)	**0.057**
Stage 3 and 4	7 (30.77)	16 (69.23)	2.64 (3.09–10.82)	**0.118**
Duration on ART				
< 12 months	9 (15.00)	51 (85.00)	14.70 (6.84–31.59)	**< 0.001**
12–36 months	33 (34.74)	62 (65.26)	4.87 (2.92–8.13)	**< 0.001**
> 36 months	166 (72.17)	64 (27.83)	Ref	**-**
ART Doses Missed Last Month				
< 2 doses	39 (39.80)	59 (60.20)	5.35 (3.14–9.11)	**< 0.001**
2–5 doses	12 (23.53)	39 (76.47)	11.49 (5.52–23.95)	**< 0.001**
> 5 doses	12 (24.00)	38 (76.00)	11.20 (5.37–23.37)	**< 0.001**
None	145 (77.96)	41 (22.04)	Ref	**-**
Opportunistic Infections				
Yes	31 (19.75)	126 (80.25)	14.11 (8.54–23.29)	**< 0.001**
No	177 (77.63)	51 (22.37)	Ref	**-**

*Significant p-value (< 0. 2). *Ref* Reference category, *OR* Odds Ratio, *CI* Confidence Interval

At multivariate analysis several factors were significantly associated with undernutrition (*p* < 0.05). These included Children whose caregivers had no formal education (AOR = 4. 54 (95% CI:2.75–8.48), *p* < 0.001) were 4.54 times more likely to be undernourished compared to those with secondary education. Household earning less than 200,000 UGX (AOR = 9.22 (95% CI: 3.15–26.93), *p* < 0.001) had 9.22 times higher odds of undernutrition compared to those households earning more than 200,000 UGX. Low birth weight < 2.5 kg (AOR = 3.48 (95% CI:1.38–8.76), *p* = 0.008) had 3.48 times higher odds of undernutrition compare to children with birth weight ≥ 2.5 kg. A high viral load (> 1000 copies/ml) (AOR = 7.43 (95% CI: 2.37–23.34), *p* = 0.001) increased the risk over seven times, While a CD4 count below 200 cells/mm³ AOR = 5.69 (95% CI:1.81–17.90), *p* = 0.003) was linked to nearly six times higher odds. Shorter duration (< 12 months) on ART (AOR = 7.80 (95% CI: 2.33–26.10), *p* = 0.001) was associated with almost eight times higher odds, while 12–36 months (AOR = 3.67 (95% CI:1.55–8.69), *p* = 0.003) had nearly four times higher odds. Missing ART doses was strongly linked to undernutrition, with those missing more than five doses (AOR = 8.45 (95% CI:2.32–30.74), *p* = 0.001) had over eight times higher odds. Lastly, children with opportunistic infections (AOR = 8.26 (95% CI:3.70–18.44), *p* < 0.001) had over eight times higher odds, (Table 5).

**Table 5:** Multivariate analysis of factors associated with undernutrition among HIV-positive children (*N* = 385)

Characteristic	cOR (95% CI)	*p*-value	AOR (95% CI)	*p*-value
Caregiver’s Education Level (Ref: Secondary)				
None	7.42 (3.94–13.97)	< 0.001	4.54 (2.75–8.48)	**< 0.001**
Primary	3.57 (2.20–5.79)	< 0.001	2.07 (0.89–4.82)	0.090
Residence (Ref: Urban)				
Rural	4.17 (2.72–6.39)	< 0.001	1.75 (0.81–3.79)	0.153
Monthly Family Income (UGX) (Ref: >500,000)				
< 200,000	10.86 (5.69–20.71)	< 0.001	9.22 (3.15–26.93)	**< 0.001**
200,000–500,000	2.53 (1.39–4.64)	0.003	2.35 (0.83–6.71)	0.109
Birth Weight (Ref: ≥2.5 kg)				
< 2.5 kg	3.53 (2.16–5.77)	< 0.001	3.48 (1.38–8.76)	**0.008**
Child Ever Admitted (Ref: No)				
Yes	1.79 (1.18–2.72)	0.006	0.77 (0.32–1.86)	0.562
Recent Viral Load (Ref: <40)				
< 1000	4.35 (2.64–7.18)	< 0.001	5.83 (2.43–13.98)	**< 0.001**
> 1000	10.09 (5.15–19.79)	< 0.001	7.43 (2.37–23.34)	**0.001**
CD4 Count (Ref: ≥350)				
< 200	20.53 (8.84–47.66)	< 0.001	5.69 (1.81–17.90)	**0.003**
200–349	3.87 (2.38–6.30)	< 0.001	1.46 (0.65–3.28)	0.366
HIV Clinical Stage (Ref: Stage 1)	Ref	-		
Stage 2	0.50 (0.32–1.50)	0.057	1.54 (0.48–4.95)	0.468
Stage 3 or 4	2.64 (3.09–10.82)	0.118	1.78 (0.28–11.32)	0.542
Duration on ART (Ref: >36 months)				
< 12 months	14.70 (6.84–31.59)	< 0.001	7.80 (2.33–26.10)	**0.001**
12–36 months	4.87 (2.92–8.13)	< 0.001	3.67 (1.55–8.69)	**0.003**
ART Doses Missed Last Month (Ref: None)				
< 2 doses	5.35 (3.14–9.11)	< 0.001	3.55 (1.50–8.40)	**0.004**
2–5 doses	11.49 (5.52–23.95)	< 0.001	4.50 (1.30–15.60)	**0.018**
> 5 doses	11.20 (5.37–23.37)	< 0.001	8.45 (2.32–30.74)	**0.001**
Cotrimoxazole (Ref: Yes)				
No	0.70 (0.47–1.05)	0.086	0.57 (0.26–1.25)	0.164
Opportunistic Infections (Ref: No)				
Yes	14.11 (8.54–23.29)	< 0.001	8.26 (3.70–18.44)	**< 0.001**

*Significant p-value (< 0. 05). *Ref* Reference category, *cOR* Crude Odds Ratio, *AOR* Adjusted Odds Ratio, *CI* Confidence Interval

## Discussion

### Prevalence of undernutrition among HIV-positive children

In this study, the overall prevalence of undernutrition was 45.97%, with stunting, underweight, and wasting at 36.62%, 24.42%, and 10. 91%, respectively. These results show that malnutrition is still a major problem, especially among children living with HIV, despite the availability of antiretroviral therapy (ART) and nutrition programs. The rates found in this study are very similar to those reported in Sub-Saharan Africa, where undernutrition was 46.7%, with stunting, underweight, and wasting at 35.9%, 23.0%, and 23.0% respectively. Similarly, in Tanzania, the prevalence of stunting, underweight, and wasting was reported as 36.6%, 22.1%, and 13.6%, respectively [1, 11].

The high prevalence of undernutrition in this study suggests that HIV-infected children remain vulnerable to poor nutritional outcomes, necessitating continued efforts to improve nutritional support and healthcare interventions [6]. When comparing HIV-positive and HIV-negative children. In this study, HIV-positive children had higher rates of stunting and underweight than HIV-negative children. This suggests that HIV infection increases the risk of poor nutrition. HIV weakens the immune system, which can lead to poor nutrient absorption, increased energy needs, frequent illnesses, and overall worse health, all of which contribute to undernutrition.

In contrast, a higher prevalence undernutrition was reported in Cameroon, with an overall malnutrition rate of 68.7%, including stunting, underweight and wasting at 63.6%, at 37.8%, at 18.4% Similarly in Tanzania a prevalence of 61.9%, with stunting, underweight wasting at 38.7%, 26.0%, 21.1% respectively [11, 12]. The comparatively lower prevalence of undernutrition in the present study may be attributed to differences in study settings, sample sizes, and access to healthcare services when compared to the populations studied in Cameroon and Tanzania.

In contrast, a lower prevalence of malnutrition was reported in Ghana, with an overall malnutrition rate of 13%, and stunting, underweight, and wasting rates of 28%, 16%, and 16%, Similarly, Ethiopia reported the prevalence of undernutrition, stunting, underweight, and wasting at 27%, 17.8%, 15.9%, and 10.7% respectively Additionally, in Hoima, Uganda, reported an overall prevalence of 28.6%, but did not break down the data by specific indicators such as stunting, underweight, or wasting [6, 8, 13]. These differences are likely due to variations in study settings, sample sizes, and healthcare access. The current study, which includes a broader age range (6 months to 12 years) and multiple healthcare centers, offers a more comprehensive estimate of the undernutrition prevalence in the district.

Despite the evident improvements in healthcare access, ART adherence, and the on-going provision of nutritional support, undernutrition remains a significant concern among HIV-positive children. The findings confirm that nutritional deficiencies persist, particularly in resource-limited settings where factors like poverty and food insecurity continue to exacerbate malnutrition. The persistence of high rates of stunting, underweight, and wasting underscores the need for comprehensive interventions that address both medical and socioeconomic determinants of undernutrition [1].

### Factors associated with undernutrition among HIV-positive children

Undernutrition in HIV-positive children is influenced by a range of sociodemographic, clinical, and economic factors, many of which were identified in this study. The findings indicate that caregiver education level, family income, birth weight, viral load, CD4 count, ART adherence, duration on ART, and opportunistic infections are significantly associated with undernutrition. These factors align with previous research in sub-Saharan Africa, highlighting the complex interactions between HIV, socioeconomic disparities, and child nutrition [1].

One of the strongest predictors of undernutrition in this study was caregiver education level. Children whose caregivers had no formal education were over eight times more likely to be undernourished (AOR = 8.54, *p* < 0.001). This finding is consistent with studies in Ethiopia and Nigeria, which found that maternal education significantly influences child nutrition, as educated caregivers are more likely to have better knowledge of feeding practices, hygiene, and healthcare-seeking behaviors [6, 12]. Limited education among caregivers can contribute to inadequate dietary diversity, poor infant feeding practices, and delayed healthcare access, all of which increase the risk of malnutrition. Economic factors also played a major role. Children from households earning less than 200,000 UGX per month were over nine times more likely to be undernourished (AOR = 9.22, *p* < 0.001), This is consistent with regional findings that link low income to food insecurity, poor sanitation, and limited healthcare access [9].

Household income also emerged as a significant determinant of undernutrition. Children from families earning less than 200,000 UGX per month were more than nine times more likely to be undernourished (AOR = 9.22, *p* < 0.001). This finding aligns with previous studies in East Africa, which reported that low socioeconomic status increases the risk of undernutrition due to food insecurity, inadequate access to healthcare, and poor sanitation [7]. Malnutrition in children from low-income households may result from insufficient dietary intake, frequent infections, and limited access to essential health services.

Children with low birth weight (< 2.5 kg) had nearly three and a half times higher odds of undernutrition (AOR = 3.48, *p* = 0.008) compared to those with normal birth weight. This is consistent with findings from studies in Cameroon and Ethiopia [6, 12]. Low birth weight is often associated with intrauterine growth restriction, maternal malnutrition, and preterm birth, all of which contribute to early childhood undernutrition [5].

Children with high viral loads (> 1,000 copies/ml) were more than seven times more likely to be undernourished (AOR = 7.43, *p* = 0.001), while those with viral loads between 40 and 1000 copies/ml had nearly six times higher odds (AOR = 5.83, *p* < 0.001). This finding is supported by previous research in Uganda and Tanzania, which reported that poor viral suppression leads to increased metabolic demands, chronic inflammation, and frequent opportunistic infections, all of which contribute to undernutrition [2, 11].

Similarly, CD4 count was a significant predictor of undernutrition. Children with CD4 counts below 200 cells/mm³ were nearly six times more likely to be undernourished (AOR = 5.69, *p* = 0.003), indicating that severe immunosuppression is strongly associated with malnutrition. Studies in Nigeria and Kenya have also shown that advanced HIV disease is linked to lower weight-for-age and height-for-age z-scores due to increased inflammation, nutrient depletion, and metabolic alterations [4, 13]. Poor ART adherence further contributed to undernutrition risk. Children missing more than five ART doses per month had over eight times higher odds of being undernourished (AOR = 8.45, *p* = 0.001), while those missing 2–5 doses had nearly four and a half times the odds (AOR = 4.50, *p* = 0.018). This is consistent with findings from sub-Saharan Africa showing that poor adherence undermines viral suppression and exacerbates nutritional challenges [1].

Duration on ART also mattered; children on ART for less than 12 months had nearly eight times higher odds of being undernourished (AOR = 7.80, *p* = 0.001), while those on ART for 12–36 months had nearly four times higher odds (AOR = 3.67, *p* = 0.003). These findings suggest that nutritional benefits accumulate with time on treatment [14]. Finally, Children with opportunistic infections had over eight times higher odds of being undernourished (AOR = 8.26, *p* < 0.001). This is in agreement with studies in Uganda and Ethiopia, which found that conditions such as tuberculosis, pneumonia, and chronic diarrhea significantly increase the risk of malnutrition in HIV-positive children Opportunistic infections elevate metabolic demands, reduce appetite, impair nutrient absorption, and lead to chronic weight loss, thereby increasing the severity of undernutrition [1, 6].

## Conclusion

This study highlights the high prevalence of undernutrition among HIV-positive children in Bushenyi District, with significant rates of stunting, underweight, and wasting. Sociodemographic and clinical factors, including caregiver education, household income, ART adherence, and viral load, also played critical roles in undernutrition.

### Study limitations

Cross-sectional Design, Limits causal inference between undernutrition and associated factors.

Recalls Bias–Caregiver socioeconomic data may be inaccurate. Self-reported ART Adherence may not fully reflect actual medication.

Limited Generalizability–Findings apply to Bushenyi District and may not reflect other regions.

No Longitudinal Follow-up–Lacks data on long-term nutritional trends and ART impact.

## Supplementary Information

Supplementary Material 1.

## Data Availability

Data can be obtained by contacting (corresponding author).

## Abbreviations

HIV: Human Immunodeficiency Virus
ART: Antiretroviral Therapy
STATA: Statistical Software Package
WHO: World Health Organization
BMI: Body Mass Index
KIU-TH: Kampala International University Teaching Hospital
IAH: Ishaka Adventist Hospital
BHC: Bushenyi Health Centre
KHC: Kyabugimbi Health Centre
CH: Comboni Hospital
TDF: Tenofovir
3TC: Lamivudine
DTG: Dolutegravir
ABC: Abacavir
AZT: Zidovudine
HAART: Highly Active Antiretroviral Therapy
