# Supplementary material for: Prevalence and factors associated with undernutrition among HIV-positive children aged 6 months to 12 years attending antiretroviral treatment clinics in Bushenyi District, Uganda: a cross-sectional study
Source: BMC Nutr. 2026 Mar 18;12:79. doi: 10.1186/s40795-026-01298-0 (PMC13112909; doi:10.1186/s40795-026-01298-0)
Supplement: Supplementary file 1 — Supplementary Material 1. [file 40795_2026_1298_MOESM1_ESM.docx]

**Appendix I: Questionnaire (English version)**

Date of interview: ………./………/……24

Respondent’s ID number………………………. …. Health Facility………………

**Title:** *“***Prevalence and factors associated with undernutrition among HIV positive children attending antiretroviral treatment clinics in Bushenyi district***”*

| **SECTION A: CHILD’S SOCIO -DEMOGRAPHIC DATA** |  |
| --- | --- |
| 1. Age (year/months) |  |
| 1. What is the sex of your baby? | 1. Male 2. Female |
| **SECTION B: MOTHER’S SOCIODEMOGRAPHIC DATA** |  |
| 1. Age (years) |  |
| 1. Level of education | 1. None 2. Primary 3. Secondary 4. Tertiary |
| 1. Occupation |  |
| 1. Residence | 1. Urban 2. Rural |
| 1. Number of living children: |  |
| 1. Marital status: | 1. Single 2. Married 3. Widow 4. Divorced |
| 1. Monthly income of the family | 1. Less than 200,000 2. 200,000-500, 000 3. More than 500,000 |
| **SECTION C: FATHER’S SOCIODEMOGRAPHIC DATA** |  |
| 1. Age (years) |  |
| 1. Level of education: | 1. None 2. Primary 3. Secondary 4. Tertiary |
| 1. Occupation |  |
| **SECTION D: MEDICAL FACTORS.** |  |
| 1. What was the weight of your baby at birth? |  |
| 1. Delivery place | 1. Home 2. Health facility |
| 1. Does the child have any known chronic disease? | 1. Yes 2. No |
| If yes precise |  |
| 1. Has you baby ever been admitted to hospital? | 1. Yes 2. No |
| If yes precise…………………………….. |  |
| 1. Has your baby ever been treated for any condition? | 1. Yes 2. No |
| If yes precise…………………………….. |  |
| 1. What is the child’s current weight |  |
| 1. Are the parents still alive? | 1. Yes 2. No |
| 1. What is the relationship with the caregiver |  |
| 1. What is the birth order |  |
| 1. Most recent viral load |  |
| 1. Most recent CD4 |  |
| 1. HIV clinical stage |  |
| 1. Duration since diagnosis of HIV |  |
| 1. Number of admissions in the previous 6 months |  |
| 1. ART regimen |  |
| 1. ART duration |  |
| 1. How many doses of ART medications the child missed in the last month | 1. <2 doses 2. 2-5 doses 3. >5 doses 4. None |
| 1. Reasons for missing ART doses | 1. Forgot 2. Run out of medication 3. Side effects 4. Lack of caregiver |
| 1. Are you giving cotrimoxazole to your baby? | 1. Yes 2. No |
| 1. If yes to question 33 above, how many times did you miss during last month? |  |
| 1. Opportunistic infections currently? | 1. Yes 2. No |
| 1. If yes, specify | 1. Oral ulcers 2. Oral candidiasis 3. Tb infection 4. Others….. |
| **SECTION E: FEEDING PRACTICES.** |  |
| **Breastfeeding practices** |  |
| 1. When did you initiate breastfeeding after delivery? | 1. Less than 20 minutes 2. within the 1^st^ h 3. after 1^st^ hour 4. After the 1^st^ day |
| 1. How did you feed you baby during his first 6 months? | 1. Never breastfed 2. Breast milk alone 3. Breast milk + cow milk 4. Infant formula alone 5. Formula + breast milk 6. Porridge or other foods 7. Porridge or other foods + breastfeeding |
| 1. For how long did you exclusively breastfeed your child? | 1. Never 2. <2 month 3. 2-3 months 4. 4-6 months |
| 1. Is your child still breastfeeding? | 1. Yes 2. No |
| 1. If No to question 40 above, for how long did you breastfeed? | *To specify ………...Months* |
| 1. If yes to question 40, how many times did you breastfeed during the last 24 hours? | 1. None 2. 1-2 times 3. 2-4 times 4. 5-6 times 5. 7-8 times 6. 9 times or More |
| 1. Have ever used a bottle with a nipple to feed your baby? | 1. Yes 2. No |
| **Other feeding practices indicators: MMF, MDD** |  |
| 1. When did you introduce other feedings   (Solid, semi-solid or soft foods) | 1. 0-1 month 2. 2-3 months 3. 4-5 months 4. 6-8 months |
| 1. How many times did you feed your child yesterday (Breastfeeding not counted) | 1. None 2. 1-2 times 3. 2-4 times 4. 5-6 times 5. > 6 times |
| 1. Which food did you give to your child last 24 hours (*Possible multiple ticks*) | |
| 1. Breast milk 2. Grains, roots and tubers 3. pulses (beans, peas,), nuts and seeds 4. Dairy products (milk, infant formula, yogurt, cheese) 5. Flesh foods (meat, fish, organ meats) 6. Eggs 7. Vitamin-A rich fruits and vegetables 8. Other fruits and vegetables | |
| Have you ever benefited from nutritional counselling  On how to feed your baby? | 1. Yes 2. No |
| **SECTION F: NUTRITIONAL ASSESSMENT** | |
| \| 1. **Bilateral pitting** \| 1). Absent 2). Grade 1 3). Grade 2 4). Grade 3 \| \| --- \| --- \| | |
| 1. **Anthropometry** | |
| \| 1. Weight ……………… Kg 2. Height/Length………. Cm 3. Age ……………...Months 4. BMI………………… \| 1. WLZ: < -3SD ≥ -3SD to < -2SD ≥ -2SD 2. LAZ: < -3SD ≥ -3SD to < -2SD ≥ -2SD 3. WAZ : < -3SD ≥ -3SD to < -2SD ≥ -2SD 4. BMI for age < -3SD ≥ -3SD -< -2SD ≥ -2SD \| \| --- \| --- \| | |
